# Reduced serum calcium is associated with a higher risk of retinopathy in non-diabetic individuals: The Chinese Multi-provincial Cohort Study

**DOI:** 10.3389/fendo.2022.973078

**Published:** 2022-11-30

**Authors:** Jiangtao Li, Dong Zhao, Qiuju Deng, Yongchen Hao, Miao Wang, Jiayi Sun, Jun Liu, Guandi Ren, Huiqi Li, Yue Qi, Jing Liu

**Affiliations:** ^1^ Center for Clinical and Epidemiologic Research, Beijing Anzhen Hospital, Capital Medical University, Beijing Institute of Heart, Lung and Blood Vessel Diseases, Beijing, China; ^2^ The Key Laboratory of Remodeling-Related Cardiovascular Diseases, Ministry of Education, Beijing, China; ^3^ Beijing Municipal Key laboratory of Clinical Epidemiology, Beijing, China; ^4^ School of Information and Electronics, Beijing Institute of Technology, Beijing, China

**Keywords:** serum calcium, retinopathy, microvascular disease, non-diabetic, convolutional neural network

## Abstract

**Aims:**

As a common micro-vascular disease, retinopathy can also present in non-diabetic individuals and increase the risk of clinical cardiovascular disease. Understanding the relationship between serum calcium and retinopathy would contribute to etiological study and disease prevention.

**Methods:**

A total of 1836 participants (aged 55–84 years and diabetes-free) from the Chinese Multi-Provincial Cohort Study-Beijing Project in 2012 were included for analyzing the relation between serum calcium level and retinopathy prevalence. Of these, 1407 non-diabetic participants with data on serum calcium in both the 2007 and 2012 surveys were included for analyzing the association of five-year changes in serum calcium with retinopathy risk. The retinopathy was determined from retinal images by ophthalmologists and a computer-aided system using convolutional neural network (CNN). The association between serum calcium and retinopathy risk was assessed by multivariate logistic regression.

**Results:**

Among the 1836 participants (male, 42.5%), 330 (18.0%) had retinopathy determined by CNN. After multivariate adjustment, the odds ratio (OR) comparing the lowest quartiles (serum calcium < 2.38 mmol/L) to the highest quartiles (serum calcium ≥ 2.50 mmol/L) for the prevalence of retinopathy determined by CNN was 1.58 (95% confidence interval [CI]: 1.10 – 2.27). The findings were consistent with the result discerned by ophthalmologists, and either by CNN or ophthalmologists. These relationships are preserved even in those without metabolic risk factors, including hypertension, high hemoglobin A1c, high fasting blood glucose, or high low-density lipoprotein cholesterol. Over 5 years, participants with the sustainably low levels of serum calcium (OR: 1.58; 95%CI: 1.05 – 2.39) and those who experienced a decrease in serum calcium (OR: 1.56; 95%CI: 1.04 – 2.35) had an increased risk of retinopathy than those with the sustainably high level of serum calcium.

**Conclusions:**

Reduced serum calcium was independently associated with an increased risk of retinopathy in non-diabetic individuals. Moreover, reduction of serum calcium could further increase the risk of retinopathy even in the absence of hypertension, high glucose, or high cholesterol. This study suggested that maintaining a high level of serum calcium may be recommended for reducing the growing burden of retinopathy. Further large prospective studies will allow more detailed information.

## Introduction

Retinopathy is generally considered to be a clinical presentation of diabetes mellitus, commonly termed diabetic retinopathy, which is the leading cause of vision impairment and blindness in working-aged people globally (1). However, typical retinopathy lesions, such as microaneurysms and retinal hemorrhages, can also be seen in middle-aged and elderly adults without diabetes, with a prevalence rate of up to 10% (2). As a common micro-vascular disease, recent studies have suggested that retinopathy could significantly increase the risk of clinical cardiovascular disease in non-diabetic individuals (2). Although hyperglycemia had been a target in patients with diabetes, there were no effective prevention and treatment approach to reduce the growing burden of retinopathy in non-diabetic individuals. Thus, identifying novel risk factors related to retinopathy is of utmost importance to understand the etiology and prevent the disease burden.

Calcium, the most abundant metallic element in the human body, is an important component of bone and teeth that participates in many important biological processes, including cell metabolism and neurotransmission (3). A recent single-cell RNA sequencing study reported that abnormal calcium and other metal ion response pathways were involved in mouse diabetic retinopathy (4). A recent study in patients with type 2 diabetes mellitus suggested the relation between circulating calcium levels and vision-threatening diabetic retinopathy (5). Yet, it remains unclear whether serum calcium and its changes are associated with the risk of retinopathy in non-diabetic individuals, due to the different local microenvironments.

Automatic retinal image analysis is an important screening tool for the detection of retinopathy, which can reduce the workload of manual grading and save diagnosis cost and time (6). Convolutional neural networks (CNN) have been proved to have high sensitivity and specificity for detecting diabetic retinopathy (7), and thus we have developed a computer-aided system based on CNN with similar high accuracy (8) for automated detection of retinopathy in retinal fundus photographs from a community-based cohort study. Thereafter, this study aimed to explore the association of serum calcium level and five-year changes in calcium with the risk of retinopathy prevalence, and further investigate the joint impact of serum calcium and metabolic risk factors on the retinopathy in non-diabetic individuals.

## Materials and methods

### Study design and study population

The study population was recruited from the Chinese Multi-Provincial Cohort Study-Beijing Project. The design and selection criteria have been previously described (9, 10). A total of 1941 participants without diabetes who participated in the examinations on demographic characteristics and traditional risk factors in 2012 were included in this study. Retinal images were collected from 1901 participants (97.9%). After excluding those with incomplete data (n=65), 1836 participants were included for analyzing the relation between serum calcium level and retinopathy prevalence. Of these, 1407 non-diabetic participants with data on serum calcium in both the 2007 survey and the 2012 survey were included for analyzing the association of five-year changes in serum calcium with retinopathy (**Figure S1**).

The study was approved by the Ethics Committee of Beijing An Zhen Hospital, Capital Medical University, and written informed consent was obtained from all participants. All research adhered to the tenets of the Declaration of Helsinki.

### Measurement of serum calcium

Serum levels of total calcium were determined by Arsenazo III colorimetry (Beckman Coulter, Brea, America) using fresh samples on the day of collection. This assay has a functional sensitivity of 0.01 mmol/L. The mean coefficient of variation was 0.57%. Albumin levels were tested by the bromocresol green colorimetric method (Beckman Coulter, Brea, America), and the mean coefficient of variation was 1.30%. Serum levels of total calcium and albumin were measured using the same method in both the 2007 and 2012 surveys. The levels of albumin-corrected calcium were calculated according to the following formula (11): *albumin-corrected calcium (mmol/L) = measured total calcium (mmol/L) + 0.02 × [40* – *albumin (g/L)]*.

### Retinal photography and definition of retinopathy

A standardized ophthalmologic examination was performed by a trained ophthalmologist. After 5 minutes of dark adaptation, macular-centered 45° digital retinal photographs of each eye were obtained using a color fundus camera (Canon EOS) without pharmacological mydriasis. Images were saved in JPEG format with a resolution of 3888×2592 pixels. Retinopathy was determined by a trained ophthalmologist in the ocular examination and defined as the presence of any lesions of micro hemangioma, hemorrhage, exudation, cotton spots, and neovascularization.

**Figure 1:**
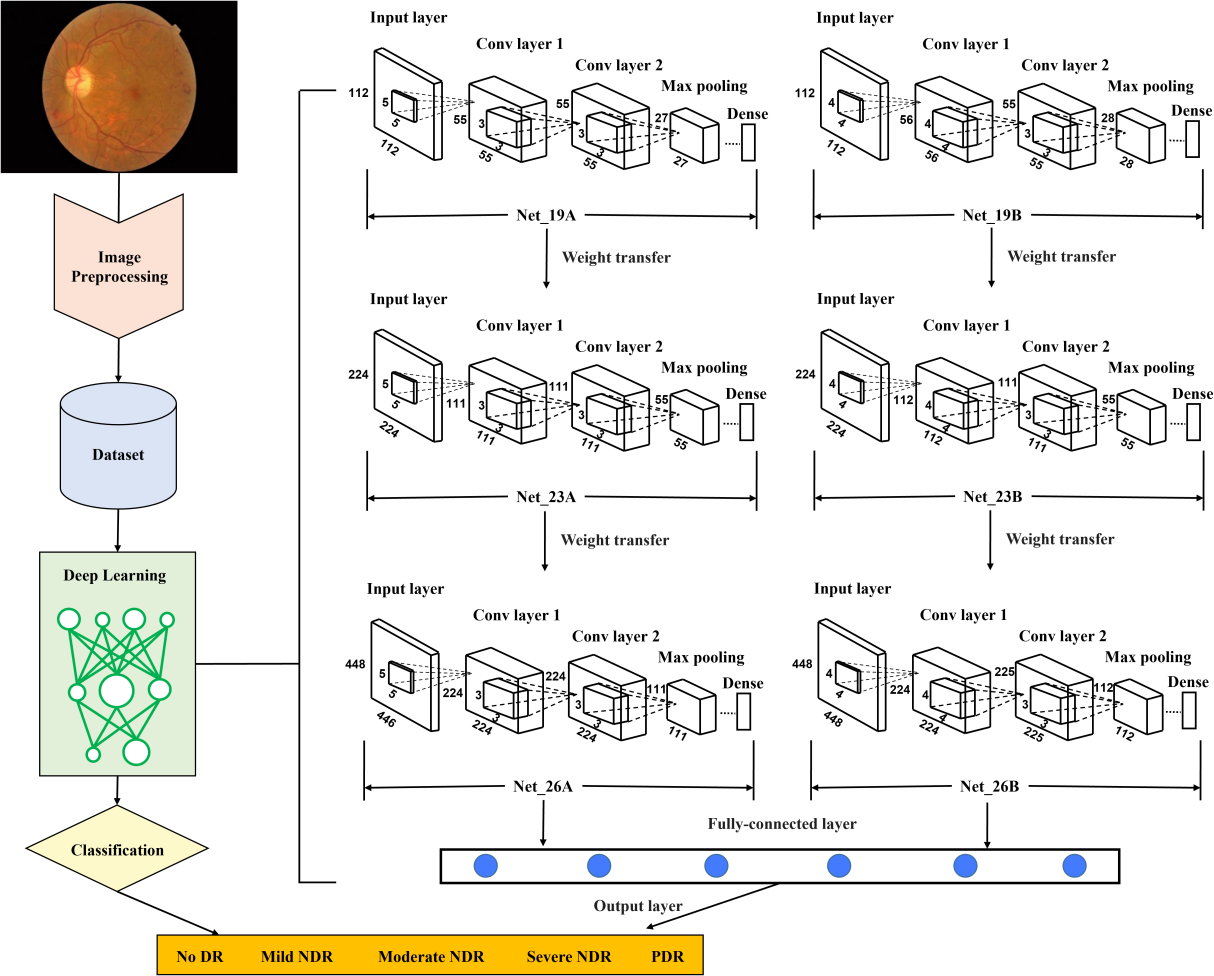
Flow diagram of the proposed retinopathy grading system. Conv, convolutional; CNN, convolutional neural network; DR, diabetic retinopathy; NDR, nonproliferative diabetic retinopathy; PDR, proliferative diabetic retinopathy. First, in the preprocessing stage, the retinal images as the training set were re-cut, and the training set was expanded to balance the proportion of five grades of images. In addition, the resolution of images was adjusted, and the preprocessed images will be unified into three different resolutions for training CNN with different layers. Second, images with a resolution of 112×112 were used to train two CNNs with 19 layers, Net_19A and Net_19B. Using the transfer learning method, assign the weights of the first eleven layers obtained by its training to the corresponding first eleven layers in the two CNNs with 23 layers, Net_23A and Net_23B. Images with a resolution of 224×224 were used to train Net_23A and Net_23B. The weights of the first fifteen layers obtained after training are assigned to the first fifteen layers of the two CNNs with 26 layers, Net_26A and Net_26B. Then images with a resolution of 448×448 were used to train Net_26A and Net_26B. Third, after all the training of the CNN was completed, the output features of the last pooling layer in the two network models of Net_26A and Net_26B were extracted and saved. Finally, the extracted features were used as input data to train the final fully connected network model.

Furthermore, a computer-aided system based on CNN was developed to detect retinopathy and lesion area marking. The design and methods have been previously described (8). Briefly, 35 092 retinal images were obtained from the Kaggle database. The images were already classified into five grades (grades 0: no apparent retinopathy; grade 1: mild nonproliferative diabetic retinopathy; grade 2: moderate nonproliferative diabetic retinopathy; grade 3: severe nonproliferative diabetic retinopathy; grade 4: proliferative diabetic retinopathy) using international clinical diabetic retinopathy severity scales (12) as the reference standard. At first, two CNNs were constructed and trained as a weak learner. The main difference between the two network models was that the convolution kernels had different sizes. Next, the features of the last pooling layers were extracted repeatedly using different data augmentation. The mean and standard deviation of the features were stored as the input of a strong learner; the output of the strong learner was the final classification results (**Figure 1**). In the test set, the classification accuracy was 80%, and the kappa value was 84%. In the present study, retinopathy grade was obtained from all the retinal images using this computer-aid system, and retinopathy was defined as grades 1–4 in any retinal images of the participant. Retinopathy severity was defined as the maximum grade in all images of one participant. In the sensitivity analyses, retinopathy was also defined as meeting any diagnostic criteria of retinopathy of CNN or ophthalmologist.

### Assessment of relevant covariates

Information on demographic characteristics, lifestyle factors, and personal medical history were collected using a standardized questionnaire. Anthropometric measurements, including height, weight, and blood pressure, were obtained by trained physicians during physical examination. Body mass index (BMI) was calculated according to the following formula: weight in kilograms divided by height in meters squared. Blood pressure was calculated by averaging three consecutive recordings, measured at the right-side brachial artery with the participants in a sitting position using a mercury sphygmomanometer after resting for at least 5 minutes. Hypertension was defined as systolic blood pressure ≥ 140 mmHg, diastolic blood pressure ≥ 90 mmHg, and/or antihypertensive treatment in the last two weeks. Smoking was defined as smoking one or more cigarettes per day for more than 3 months. Diabetes was determined as any of the following criteria (1): fasting blood glucose (FBG) ≥ 7.1mmol/L (2), hemoglobin A1c (HbA1c) ≥ 6.5% (only in the 2012 survey) (3), the previous diagnosis by a physician, or (4) use of insulin or glucose-lowering medications during the past month. History of cardiovascular disease were defined as presence of coronary heart disease or stroke events.

Laboratory measurements were performed by using fresh samples on the day of collection. FBG levels were measured using enzymatic methods (Beckman Coulter, Brea, America). HbA1c levels were determined using ion-exchange high-performance liquid chromatography (BIO-RAD Turbo, Hercules, America). Triglyceride and total cholesterol levels were tested by enzymatic methods (Sekisui Medical Co., Ltd, Tokyo, Japan). High-density lipoprotein cholesterol (HDL-C) and low-density lipoprotein cholesterol (LDL-C) levels were measured using a homogeneous method (Sekisui Medical Co., Ltd, Tokyo, Japan). High-sensitivity C reactive protein (hs-CRP) levels were analyzed using immunoturbidimetric methods (Diasys Diagnostic Systems (Shanghai) Co., Ltd, Shanghai, China). Creatinine levels were measured using the enzymic method (Sekisui Medical Co., Ltd, Tokyo, Japan). Magnesium levels were tested by the dimethyl aniline blue colorimetric method (Beckman Coulter, Brea, America). The estimated glomerular filtration rate (eGFR) was calculated by the following formula (13): *eGFR (mL/min/1.73m^2^) = 186 ×creatinine (mg/dL) ^– 1.154^ × age ^– 0.203^ × 1.233 (× 0.742 in females)*.

### Sample power estimation

Until now, no studies have investigated the association between serum calcium and retinopathy prevalence in non-diabetic adults. In the present study, the prevalence of retinopathy was 18.0%, and the R-squared value for serum calcium with other covariates was 0.201. The risk estimate of serum calcium for retinopathy was 1.58. Assuming an alpha (probability of type I error) of 0.05, the actual sample size of 1836 enabled sufficient statistical power (power = 0.87).

### Statistical analysis

Participants’ characteristics were described using percentages for categorical variables and mean ± standard deviation (SD) or medians (interquartile range) for continuous variables across quartiles of serum calcium (cutoff points: 2.38, 2.44, and 2.50 mmol/L). Categorical variables were compared using the chi-square test and continuous variables using the student *t*-test or Wilcoxon rank-sum test. Normality was analyzed by Kolmogorov- Smirnov test **(** **Table S1** **)**. Correlations between the levels of serum calcium and other covariate were estimated using partial correlations controlling for age and sex. *P* for trend was test by binary logistic regression for categorical variables and linear regression for continuous variables, and quartiles of serum calcium levels were included in the models as the independent variable.

Odds ratio (OR) for the risk of retinopathy prevalence associated with serum calcium levels were calculated using the binary logistic regression model controlling for traditional risk factors of retinopathy and variables associated with calcium levels, including: age (per 1 years), sex, BMI (per 1 kg/m^2^), smoking, systolic blood pressure (< 140 mmHg, 140–159 and ≥ 160 mmHg), antihypertensive treatment, HDL-C (≥ 1.04 [male]/1.30 [female] mmol/L and < 1.04 [male]/1.30 [female] mmol/L), LDL-C (< 3.4 mmol/L and ≥ 3.4 mmol/L), natural log-transformed triglyceride, lipid-lowering treatment, natural log-transformed hs-CRP, albumin (per 1 g/L), eGFR (per 1 mL/min/1.73m^2^), serum magnesium (per 0.1 mmol/L decrease), and FBG (< 5.19 [median] mmol/L and ≥ 5.19 mmol/L) or HbA1c (< 5.6% [median] and ≥ 5.6%). Serum calcium levels as a continuous variable (per SD decrease) and a categorical variable (the highest quartile of calcium as the reference) were included in the analyses, respectively.

To investigate the dose-response relationship between retinopathy prevalence risk and serum calcium as a continuous variable, the restricted cubic splines in logistic regression were performed, with three knots (10th, 50th, and 90th percentiles of calcium) recommended according to Loic Desquilbet and FrançoisMariotti (14, 15) and the first quartile of calcium (2.38 mmol/L) as the reference.

Subgroup analyses were performed using traditional risk factors, including age (< 65 years, ≥ 65 years), sex, BMI (< 24 kg/m^2^, ≥ 24 kg/m^2^), smoking, hypertension, hs-CRP (< 1mg/L, ≥ 1 mg/L), HDL-C (< 1.04 [male]/1.30 [female] mmol/L, ≥ 1.04 [male]/1.30 [female] mmol/L), LDL-C (< 3.4 mmol/L, ≥ 3.4 mmol/L), triglyceride (< 1.7 mmol/L, ≥ 1.7 mmol/L), lipid-lowering treatment, albumin (< 45g/L [median], ≥ 45g/L), HbA1c (< 5.6% [median], ≥ 5.6%), FBG (< 5.19 mmol/L [median], ≥ 5.19 mmol/L), and serum magnesium (< 0.92 mmol/L [median], ≥ 0.92 mmol/L). Serum calcium levels as a categorical variable (< 2.38 mmol/L *vs*. ≥ 2.38 mmol/L) were included in the analyses separately. Beside the grouping factors, other factors were used as covariates. *P* for interaction was assessed by including a multiplicative interaction term in the logistic regression models to estimate the interaction on retinopathy between calcium and subgroup variables.

Several sensitivity analyses were performed. First, the association between serum calcium levels and retinopathy defined by the ophthalmologist, and by CNN or ophthalmologist was investigated. Second, participants with pre-diabetes (FBG ≥ 6.1 mmol/L) or those with cardiovascular disease and whose calcium levels were not within the normal reference range (2.25–2.75 mmol/L) were excluded. Third, total serum calcium was substituted with albumin-corrected calcium for analyzing the association with retinopathy, as it may better reflect the physiological state of calcium in the body (16). Fourth, the association between serum calcium and retinopathy risk was also assessed by a propensity score-based inverse probability of treatment weighting (IPTW) method. The propensity score (ps) was calculated using a logistic regression model in which the level of serum calcium (< 2.38 mmol/L vs. ≥ 2.38 mmol/L) was the dependent variable and other covariates included age, sex, BMI, smoking, systolic blood pressure, antihypertensive treatment, HDL-C, LDL-C, natural log-transformed triglyceride, lipid-lowering treatment, natural log-transformed hs-CRP, albumin, eGFR, magnesium, and FBG. The IPTWs were 1/ps and 1/(1-ps) for calcium < 2.38 mmol/L and ≥ 2.38 mmol/L respectively. Group differences were evaluated by standardized mean differences (SMD), with SMDs ≤0.10 indicating balance characteristics. The SMD was calculated with IPTW unweighted and weighted (**Figure S2**). In the IPTW model, only calcium level was included, and the model was weighted by IPTW.

Joint analysis for the association of serum calcium and metabolic risk factors with retinopathy was performed by dividing participants into four groups according to calcium levels and metabolic risk factors. Participants with calcium ≥ 2.38 mmol/L and without metabolic risk factors as the reference group. Metabolic risk factors in the present study included high levels of HbA1c (≥ 5.6%), FBG (≥ 5.19 mmol/L), LDL-C (≥ 3.4 mmol/L), and hypertension.

To explore the association of five-year changes in serum calcium with retinopathy, participants were divided into the following four groups: maintaining a high level of serum calcium during five years (serum calcium ≥ 2.34 mmol/L [first quartile] in the 2007 survey and ≥ 2.38 mmol/L [first quartile] in 2012 survey), serum calcium decreased from high level to low level during five years (serum calcium ≥2.34 mmol/L in 2007 survey and <2.38 mmol/L in 2012 survey), serum calcium increased from low level to high level during five years (serum calcium <2.34 mmol/L in 2007 survey and ≥2.38 mmol/L in 2012 survey), and maintaining a low level of serum calcium during five years (serum calcium <2.34 mmol/L in 2007 survey and <2.38 mmol/L in 2012 survey). The first group was defined as the reference group, and binary logistic regression models were performed to calculate the ORs of the other three groups for the retinopathy risk by controlling for age, sex, BMI, smoking, systolic blood pressure, antihypertensive treatment, HDL-C, LDL-C, natural log-transformed triglyceride, lipid-lowering treatment, natural log-transformed hs-CRP, albumin, eGFR, serum magnesium, and FBG.

To investigate whether missing data would lead to potential bias, comparisons were performed between participants included in the study and those excluded due to incomplete data (**Tables S2, S3**). No significant differences in general characteristics were observed.

All statistical analyses were performed using the R software (version 3.6.2, R Foundation for Statistical Computing). A *P* value of < 0.05 on the two-sided test was considered statistically significant. Sample size estimation was calculated using the PASS software (version 11.0, NCSS, Kaysville, UT).

## Results

### General characteristics of the participants

Among 1836 non-diabetic participants, the mean age was 66.4 ( ± 7.8) years, and 42.5% were males. The mean (range) level of serum calcium was 2.44 (2.06, 2.85) mmol/L, with an SD of 0.11. The mean (range) level of albumin-corrected calcium was 2.34 (1.93, 2.71) mmol/L. The participants’ characteristics, when stratified into serum calcium quartile, are shown in **Table 1**. Statistically significant trends were found for all known cardiovascular risk factors (all *P* < 0.05), except for eGFR and age. Participants with a lower level of serum calcium were more likely to be smokers. In addition, individuals with the lowest quartiles of serum calcium had higher BMI and hs-CRP, but lower blood pressure, lipids, glucose, and rates of related treatments. Weak correlations were found between serum calcium and cardiovascular risk factors after controlling for age and sex (**Figure S3**).

**Table 1:** The characteristics of participants across the quartiles of serum calcium.

Characteristic ^a^	Quartiles of serum calcium (mmol/L)				*P* for trend
	<2.38 (n = 435)	2.38~2.44 (n = 457)	2.44~2.50 (n = 434)	≥2.50 (n = 510)	
Calcium, mmol/L	2.32 ± 0.04	2.41 ± 0.02	2.46 ± 0.02	2.56 ± 0.06	< 0.001
Albumin-corrected calcium, mmol/L	2.24 ± 0.05	2.30 ± 0.05	2.36 ± 0.05	2.43 ± 0.06	< 0.001
Age, years	64 (59, 74)	64 (59, 72)	67 (59, 74)	66 (59, 73)	0.104
Male, n (%)	227 (52.2)	187 (40.9)	183 (42.2)	184 (36.1)	< 0.001
Body mass index, kg/m^2^	24.7 (22.6, 27.1)	24.3 (22.4, 26.7)	23.8 (22.0, 26.1)	23.9 (21.9, 26.1)	< 0.001
Smoking, n (%)	64 (14.7)	59 (12.9)	49 (11.3)	41 (8.0)	0.001
Systolic blood pressure, mmHg	135.3 (125.3, 147.7)	135.0 (124.5, 145.0)	135.3 (125.3, 145.3)	138.0 (128.0, 148.3)	0.005
Diastolic blood pressure, mmHg	77.3 (72.3, 84.7)	79.0 (72.7, 84.7)	78.2 (72.3, 84.7)	80.3 (73.3, 85.8)	0.017
Hypertension, n (%)	245 (56.3)	248 (54.3)	249 (57.4)	328 (64.3)	0.006
Antihypertensive treatment, n (%)	163 (37.5)	161 (35.2)	164 (37.8)	240 (47.1)	< 0.001
Fasting blood glucose, mmol/L	5.08 (4.81, 5.42)	5.16 (4.87, 5.50)	5.22 (4.90, 5.54)	5.30 (4.97, 5.63)	< 0.001
Hemoglobin A1c, %	5.6 (5.4, 5.8)	5.7 (5.4, 5.9)	5.7 (5.4, 5.9)	5.7 (5.4, 5.9)	0.010
Pre-diabetes, n (%)	27 (6.2)	29 (6.3)	22 (5.1)	52 (10.2)	0.031
Total cholesterol, mmol/L	5.09 ± 0.95	5.31 ± 1.01	5.27 ± 1.01	5.44 ± 1.09	< 0.001
LDL-C, mmol/L	2.98 ± 0.81	3.11 ± 0.88	3.07 ± 0.81	3.18 ± 0.92	0.001
HDL-C, mmol/L	1.27 (1.08, 1.47)	1.28 (1.11, 1.49)	1.33 (1.13, 1.56)	1.37 (1.16, 1.59)	< 0.001
Triglyceride, mmol/L	1.25 (0.87, 1.75)	1.37 (0.97, 1.98)	1.37 (0.98, 1.85)	1.40 (1.01, 2.02)	< 0.001
Lipid-lowering treatment, n (%)	62 (14.3)	83 (18.2)	113 (26.0)	142 (27.8)	< 0.001
Hs-CRP, mg/L	1.16 (0.59, 2.52)	0.99 (0.52, 1.94)	0.85 (0.43, 1.76)	0.78 (0.39, 1.59)	< 0.001
Albumin, g/L	44.31 ± 2.24	45.2 ± 2.29	45.31 ± 2.34	46.25 ± 2.26	< 0.001
eGFR, mL/min/1.73m^2^	109.40 (96.14, 121.99)	108.75 (95.86, 119.79)	105.22 (95.74, 119.59)	105.42 (92.80, 117.82)	0.131
Magnesium, mmol/L	1.27 (1.08, 1.47)	1.28 (1.11, 1.49)	1.33 (1.13, 1.56)	1.37 (1.16, 1.59)	0.026
History of cardiovascular disease, n (%)	32 (7.4)	33 (7.2)	31 (7.1)	35 (6.9)	<0.001

eGFR, the estimated glomerular filtration rate; HDL-C, high-density lipoprotein cholesterol; hs-CRP, high-sensitivity C reactive protein; LDL-C, low-density lipoprotein cholesterol.

a Data are expressed as mean (standard deviation) for continuous variables in the case of normal distributions and median (interquartile range); otherwise, as number (percent) for categorical variables.

### The association of serum calcium with retinopathy

**Figure 2:**
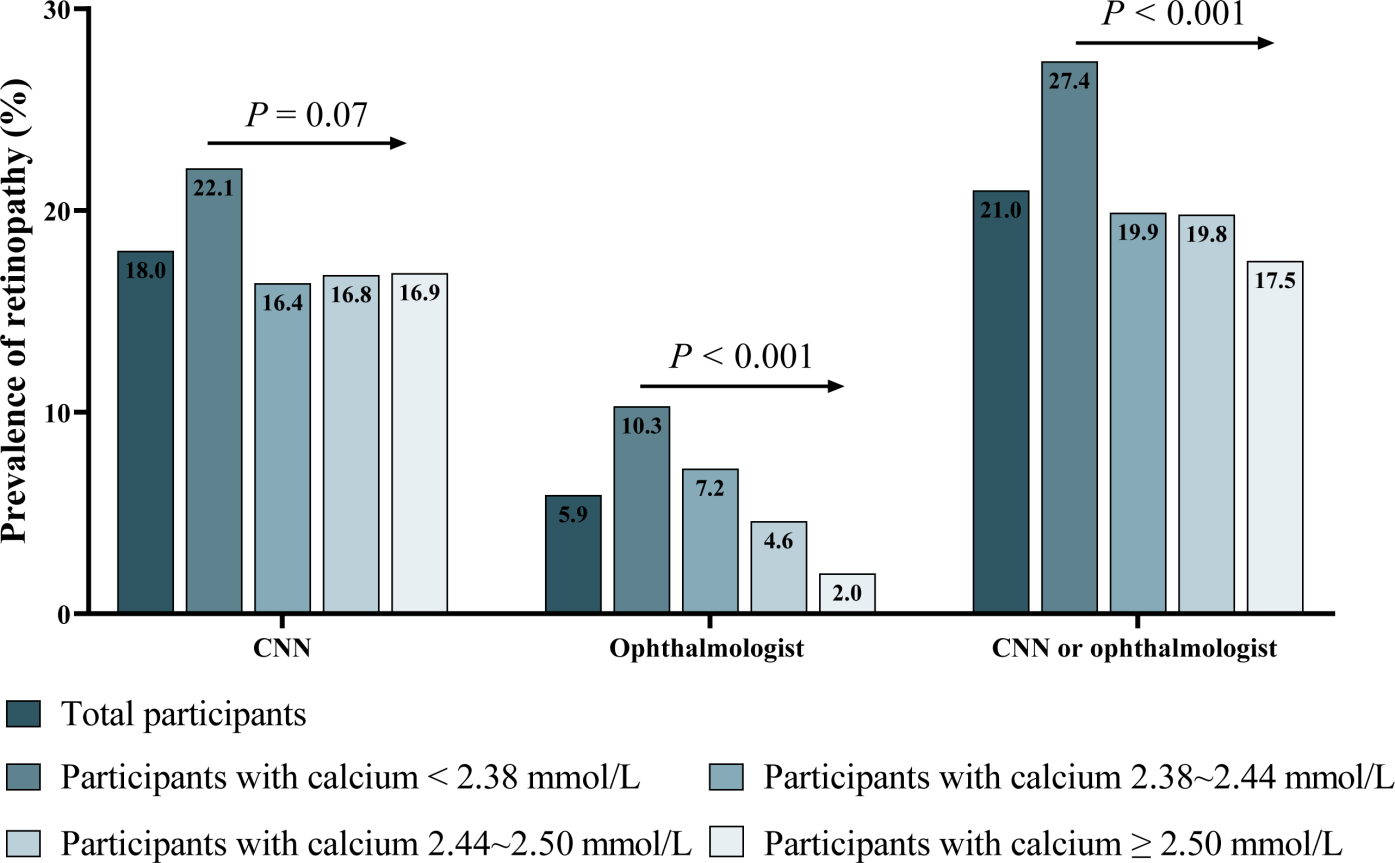
Prevalence of retinopathy discerned by CNN, ophthalmologist, and by either CNN or ophthalmologist. CNN, convolutional neural network. *P*: *P* for trend tested by chi-square test for trend.

The prevalence of retinopathy determined by CNN and ophthalmologists was 18.0% and 5.9% in non-diabetic participants, respectively (**Figure 2**). Serum calcium was negatively associated with retinopathy prevalence (**Figure 2** and **Figure S4**). After multivariate adjustment, participants with the lowest quartiles (<2.38mmol/L) of serum calcium had a significantly higher risk for the prevalence of retinopathy discerned by CNN (OR: 1.58; 95% confidence interval [CI]: 1.10-2.27; *P* =0.015) than those with the highest quartiles (**Table 2** and **Table S4**). The ORs remained unchanged after the adjustment of HbA1c substituting for FBG. These findings were consistent with data provided by the ophthalmologist, and by either CNN or the ophthalmologist (**Table 2**). The relationship between serum calcium and retinopathy prevalence remained significant after excluding participants with pre-diabetes, history of cardiovascular disease, or those without a normal reference range of serum calcium (**Table S5** **)**, as well as substituting total calcium for albumin-corrected calcium (**Table S6**). In a IPTW sample, participants with calcium < 2.38 mmol/L had a 56% increased risk of retinopathy compared to those with calcium ≥ 2.38 mmol/L (OR: 1.56; 95% CI: 1.32-1.84; *P <*0.001).

**Table 2:** Logistic regression analyses of serum calcium quartiles and per SD decrease with retinopathy.

Diagnostic methods	Calcium (mmol/L)	Unadjusted	Model 1	Model 2
CNN	≥2.50	Reference	Reference	Reference
	2.44-2.50	1.00 (0.71, 1.40)	1.05 (0.74, 1.50)	1.05 (0.74, 1.49)
	2.38-2.44	0.97 (0.69, 1.36)	1.09 (0.76, 1.56)	1.08 (0.75, 1.54)
	<2.38	1.40 (1.01, 1.93)	1.58 (1.10, 2.27)	1.55 (1.08, 2.23)
	Per SD	1.14 (1.00, 1.29)	1.20 (1.04, 1.38)	1.19 (1.03, 1.37)
Ophthalmologist	≥2.50	Reference	Reference	Reference
	2.44-2.50	2.42 (1.12, 5.22)	2.85 (1.30, 6.26)	2.80 (1.28, 6.14)
	2.38-2.44	3.89 (1.90, 7.99)	5.05 (2.39, 10.66)	4.89 (2.32, 10.31)
	<2.38	5.77 (2.87, 11.59)	8.48 (4.00, 18.02)	8.14 (3.84, 17.25)
	Per SD	1.89 (1.52, 2.35)	2.30 (1.77, 2.97)	2.26 (1.74, 2.92)
CNN or ophthalmologist ^a^	≥2.50	Reference	Reference	Reference
	2.44-2.50	1.17 (0.84, 1.62)	1.28 (0.91, 1.79)	1.27 (0.90, 1.78)
	2.38-2.44	1.18 (0.85, 1.63)	1.39 (0.99, 1.96)	1.37 (0.97, 1.93)
	<2.38	1.78 (1.31, 2.43)	2.22 (1.56, 3.14)	2.17 (1.53, 3.07)
	Per SD	1.26 (1.11, 1.42)	1.39 (1.21, 1.60)	1.38 (1.20, 1.58)

CNN, convolutional neural network; SD, standard deviation; OR, odds ratio.

a Retinopathy is defined as meeting any diagnostic criteria of retinopathy of CNN or ophthalmologist. Model 1: adjusted for age, sex, body mass index, smoking, systolic blood pressure, antihypertensive treatment, high-density lipoprotein cholesterol, low-density lipoprotein cholesterol, natural log-transformed triglyceride, lipid-lowering treatment, fasting blood glucose, natural log-transformed high-sensitivity C-reactive protein, albumin, the estimated glomerular filtration rate, and magnesium. Model 2: model 1 + hemoglobin A1c substituting for fasting blood glucose.

No differences were found between serum calcium and retinopathy prevalence among various subgroups (**Figure S5**). Further joint analysis showed that a low level of serum calcium exaggerated the impact of high glucose or high cholesterol on retinopathy risk. Compared with participants with HbA1c <5.6% and calcium ≥2.38 mmol/L, participants with HbA1c level ≥5.6% but serum calcium <2.38 mmol/L had 62% higher risk for retinopathy (OR: 1.62; 95%CI: 1.09-2.40; *P* =0.023). However, participants with HbA1c ≥5.6% and serum calcium ≥2.38 mmol/L had a comparable risk as those with a low level of HbA1c (<5.6%) but the same level of serum calcium (**Figure S6**). Similar results were found when analyzing FBG and LDL-C.

### The association of five-year changes in serum calcium with retinopathy

Changes in serum calcium over 5 years and their association with the risk of retinopathy prevalence were further investigated among participants without diabetes over 5 years. There are 11.8% of participants maintained a low level of serum calcium over 5 years, and they had a 58% increased risk for retinopathy discerned by CNN (OR: 1.58; 95%CI: 1.05-2.39, *P* =0.030), compared with those who maintained their serum calcium at a high level. Another 12.4% of participants experienced a decrease in serum calcium from high level to low level over the period, and they had a 56% increased risk (OR: 1.56; 95%CI: 1.04-2.35, *P* =0.033). However, participants whose serum calcium increased from low level to high level over 5 years did not have a heightened risk for retinopathy (OR: 1.27; 95%CI: 0.82-1.97, *P* =0.278) (**Table 3**). Similar results were found in the association of changes in serum calcium with retinopathy discerned by the ophthalmologist, and by either CNN or the ophthalmologist.

**Table 3:** The association between five-year changes in serum calcium and retinopathy.

The five-year changes in serum calcium levels	n/N (%)	OR (95%CI)^a^	*P*
Retinopathy discerned by CNN			
Sustainably high-level^b^	155/912 (17.0)	Reference	
Decreased from high level to low level^c^	41/174 (23.6)	1.56 (1.04, 2.35)	0.033
Increased from low level to high level^d^	31/154 (20.1)	1.27 (0.82, 1.97)	0.278
Sustainably low level^e^	41/167 (24.0)	1.58 (1.05, 2.39)	0.030
Retinopathy discerned by the ophthalmologist			
Sustainably high-level^b^	41/912 (4.5)	Reference	
Decreased from high level to low level^c^	17/174 (9.8)	2.62 (1.41, 4.88)	0.002
Increased from low level to high level^d^	10/154 (6.5)	1.52 (0.74, 3.13)	0.254
Sustainably low level^e^	24/167 (14.4)	4.14 (2.35, 7.32)	<0.001
Retinopathy discerned by either CNN or the ophthalmologist			
Sustainably high-level^b^	177/912 (19.4)	Reference	
Decreased from high level to low level^c^	48/174 (27.6)	1.71 (1.16, 2.53)	0.007
Increased from low level to high level^d^	35/154 (22.7)	1.27 (0.83, 1.92)	0.268
Sustainably low level^e^	54/167 (32.3)	2.14 (1.46, 3.14)	<0.001

CI, confidence interval; CNN, convolutional neural network; N, number; OR, odds ratio.

a ORs were calculated by logistic regressions after adjusting for age, sex, body mass index, smoking, systolic blood pressure, antihypertensive treatment, high-density lipoprotein cholesterol, low-density lipoprotein cholesterol, natural log-transformed triglyceride, lipid-lowering treatment, fasting blood glucose, natural log-transformed high-sensitivity C-reactive protein, albumin, the estimated glomerular filtration rate, and serum magnesium.

b Serum calcium ≥2.34 mmol/L (first quartile) in the 2007 survey and ≥2.38 mmol/L (first quartile) in the 2012 survey.

c Serum calcium ≥2.34 mmol/L in the 2007 survey and <2.38 mmol/L in the 2012 survey.

d Serum calcium <2.34 mmol/L in the 2007 survey and ≥2.38 mmol/L in the 2012 survey.

e Serum calcium <2.34 mmol/L in the 2007 survey and <2.38 mmol/L in the 2012 survey.

## Discussion

In this large community-based study, we examined the association between serum calcium levels and changes with retinopathy, based on reliable and repeated measurements of serum calcium, as well as a clear diagnosis of retinopathy by the ophthalmologist and deep learning. After controlling for covariates, reduced levels of serum calcium were significantly associated with an increased risk of retinopathy prevalence among non-diabetic individuals. Moreover, these relationships are preserved even among those without hypertension, high glucose, or high cholesterol levels. These findings suggested important implications of calcium metabolism for current retinopathy management strategies. However, as the potential for confounding may exist, further studies are warranted.

Limited evidence reported the association between serum calcium and the risk of retinopathy in non-diabetic individuals, a common micro-vascular disease that may precede macrovascular disease. The present study explored the contribution of serum calcium concentrations to the risk of retinopathy and suggested that reduced serum calcium is independently associated with a higher risk of retinopathy in non-diabetic individuals, and this impact remained significant in participants without other reported influential factors separately. With the extension, our study further investigated the association between five-year changes in serum calcium and retinopathy and found that the sustainable decrease of serum calcium levels was significantly associated with an increased risk of retinopathy.

Limited evidence was found on the impact of serum calcium on retinopathy among non-diabetic adults. Calcium balance in circulation changes during life stages and is mostly determined by body calcium requirement and dietary calcium intake. Previous studies have reported that decreased serum vitamin D, as the influential factor of body calcium requirement, was associated with an increased risk of retinopathy prevalence among 5120 non-diabetic participants in the Rotterdam Study (17). The reduction of vitamin D could lead to low serum calcium levels by reducing the reabsorption of calcium by renal tubules and inhibiting the absorption of calcium by intestinal mucosal epithelium (18), suggesting the potential role of serum calcium on the risk of retinopathy in non-diabetic individuals. Meanwhile, the Blue Mountains Eye Study (19) and Age-Related Eye Disease Study (20) all found that decreased dietary calcium intake was associated with a higher risk of late age-related macular degeneration (AMD) incidence, suggesting the possibility of targeting calcium to prevent retinopathy. Previous studies found that smoking was associated with a higher risk of retinopathy (21). In our study, participants with a lower levels of serum calcium were more likely to be smokers, suggesting that the potential impact of smoking on the association between the risk of retinopathy and serum calcium levels.

However, a recent study conducted in patients with type 2 diabetes has shown the opposite findings compared to the present study among non-diabetic individuals, with a high level of serum calcium independently associated with the increased risk of diabetic retinopathy (5). Several reasons may account for the difference. First, the study patients were all from hospitals and recruited over a 10-year period, which was different from the population without diabetes recruited from the community in the present study. Second, the underlying pathogenesis of retinopathy may be different between diabetic and non-diabetic individuals (22). Third, calcium metabolism may be different in individuals with and without diabetes. Compared with non-diabetic individuals, the calcium ion metabolism of diabetic patients is in a negative balance state (23), mainly manifested as increased calcium excretion in urine. Finally, the association of high glucose or high cholesterol with retinopathy was not found in non-diabetic individuals in the present study. Reduction of serum calcium could unexpectedly exaggerate the impact of high glucose or high cholesterol on the risk of retinopathy, even at a normal level. By these findings, elevated glucose may alter calcium homeostasis in the retina (24) and enhance constriction of retinal venules through activation of the reverse-mode sodium-calcium exchanger (25). On the other hand, abnormalities in circulating calcium levels could have a crucial role in insulin release and glucose homeostasis (26). The impact of decreased calcium on the relation between high glucose and retinopathy risk warrants prospective assessment in the future.

Several *in vivo* and *in vitro* studies support the association between lower serum calcium levels and a higher risk of retinopathy. When serum calcium levels decrease, the intracellular calcium increases, which is known as “the abnormal calcium influx” (27), thus causing the vascular smooth muscle to contract, and eventually increasing vascular resistance (28). Low serum calcium levels may also stimulate the release of parathyroid hormone and renin, thereby increasing calcium in smooth muscle cells and leading to vasoconstriction (28). The increasing vascular resistance and vasoconstriction further lead to the reduction of blood flow, which may be crucial for retinopathy where blood flow regulation is disrupted. In addition, previous animal studies demonstrated that the reduction of glial calcium signaling could cause capillary contraction, thereby reducing capillary blood flow in the mouse retina (29). However, the potential mechanism between calcium and retinopathy remains unknown and requires external validation to corroborate its utility.

This study has a few limitations. First, this cross-sectional analysis from a community-based cohort study suggested a possible association between low calcium and a higher risk of retinopathy; however, the causal-effect relationship cannot be inferred. Consequently, further prospective studies are needed. Second, some potential confounding factors, which may regulate or affect serum calcium levels and changes, were unavailable, including dietary calcium, serum parathyroid hormone, serum vitamin D, several drugs (calcium salts, calcium supplements, vitamin D supplements, glucocorticosteroids, antiresorptive drugs, recombinant parathyroid hormone) (30), and the circadian variation in serum calcium (31). Third, the ionized calcium level was not measured in the present study, which represents the physiologically active fraction of serum calcium. Although total calcium levels and albumin-adjusted calcium levels have been reported to be reasonably correlated to ionized calcium levels (32, 33), further studies are needed to explore the association between retinopathy and ionized calcium levels in non-diabetic individuals.

In conclusion, our study suggested that a low level of serum calcium and its sustainably decrease over five years were independently associated with an increased risk of retinopathy prevalence in non-diabetic individuals. Moreover, reduction of serum calcium can increase the risk of retinopathy even among individuals without high glucose, high LDL-C, or hypertension. This study provides an important clue on the risk of retinopathy associated with reduced calcium and foundational data for future intervention studies, which are essential if targeting calcium is to be considered to prevent disease burden. Yet, more studies are needed to obtain detailed information and determine causal-effect relationship, and to investigate the role of altered calcium homeostasis in the pathogenesis of the microvascular disease.

## Data availability statement

The original contributions presented in the study are included in the article/**Supplementary Material**, further inquiries can be directed to the corresponding authors.

## Ethics statement

The studies involving human participants were reviewed and approved by Ethics Committee of Beijing An zhen Hospital, Capital Medical University. The patients/participants provided their written informed consent to participate in this study.

## Author contributions

DZ, QD, YH, MW, QY, and JiL designed the study. JTL, JS, JuL, GR, and HL cleaned the data. JTL analyzed the data. JTL, YQ, and JiL wrote the manuscript. All authors reviewed and edited the manuscript. All authors contributed to the article and approved the submitted version.

## Funding

This study was supported by the Beijing Natural Science Foundation (No. 7212006); the National Natural Science Foundation of China (grant numbers 82073635, 82103962,and 81570409); Beijing municipal medical research institutes pilot reform project (grant number 2021-07); the National Key Research and Development Program of China (grant number 2016YFC0900902); the National Science & Technology Pillar Program (grant numbers 2011BAI09B01, 2011BAI11B03, 2006BAI01A01, and 2006BAI01A02); and the National Key R&D Program of China (2019YFE0116000). The sponsors had no role in the design or conduct of the study.

## Conflict of interest

The authors declare that the research was conducted in the absence of any commercial or financial relationships that could be construed as a potential conflict of interest.

## Publisher’s note

All claims expressed in this article are solely those of the authors and do not necessarily represent those of their affiliated organizations, or those of the publisher, the editors and the reviewers. Any product that may be evaluated in this article, or claim that may be made by its manufacturer, is not guaranteed or endorsed by the publisher.

## Abbreviations

AMD, age-related macular degeneration; BMI, body mass index; CI, confidence interval; Conv, convolutional; CNN, convolutional neural network; DR, diabetic retinopathy; DBP, diastolic blood pressure; FBG, fasting blood glucose; HbA1c, hemoglobin A1c; HDL-C, high-density lipoprotein cholesterol; hs-CRP, high-sensitivity C reactive protein; IPTW, the inverse probability of treatment weighting; LDL-C, low-density lipoprotein cholesterol; NDR, nonproliferative diabetic retinopathy; OR, odds ratio; PDR, proliferative diabetic retinopathy; ps, propensity score; SD, standard deviation; SMD, standardized mean differences; SBP, systolic blood pressure; eGFR, the estimated glomerular filtration rate; TC, total cholesterol; TG, triglyceride.
