# Corrigendum: Reduced serum calcium is associated with a higher risk of retinopathy in non-diabetic individuals: The Chinese Multi-provincial Cohort Study

**DOI:** 10.3389/fendo.2022.1114158

**Published:** 2022-12-21

**Authors:** Jiangtao Li, Dong Zhao, Qiuju Deng, Yongchen Hao, Miao Wang, Jiayi Sun, Jun Liu, Guandi Ren, Huiqi Li, Yue Qi, Jing Liu

**Affiliations:** ^1^ Center for Clinical and Epidemiologic Research, Beijing Anzhen Hospital, Capital Medical University, Beijing Institute of Heart, Lung and Blood Vessel Diseases, Beijing, China; ^2^ The Key Laboratory of Remodeling-Related Cardiovascular Diseases, Ministry of Education, Beijing, China; ^3^ Beijing Municipal Key laboratory of Clinical Epidemiology, Beijing, China; ^4^ School of Information and Electronics, Beijing Institute of Technology, Beijing, China

**Keywords:** serum calcium, retinopathy, microvascular disease, non-diabetic, convolutional neural network

In the published article, there was an error regarding the affiliations for Jiangtao Li, Dong Zhao, Qiuju Deng, Yongchen Hao, Miao Wang, Jiayi Sun, Jun Liu, Guandi Ren, Huiqi Li, Yue Qi and Jing Liu.

As well as having affiliation(s) 1,2, Jiangtao Li, Dong Zhao, Qiuju Deng, Yongchen Hao, Miao Wang, Jiayi Sun, Jun Liu, Yue Qi and Jing Liu should also have affiliation 3. Guandi Ren and Huiqi Li should not have affiliation 3.

The authors apologize for this error and state that this does not change the scientific conclusions of the article in any way. The original article has been updated.

