# Supplementary material for: Reduced serum calcium is associated with a higher risk of retinopathy in non-diabetic individuals: The Chinese Multi-provincial Cohort Study
Source: Front Endocrinol (Lausanne). 2022 Nov 30;13:973078. doi: 10.3389/fendo.2022.973078 (PMC9747923; doi:10.3389/fendo.2022.973078)
Supplement: Supplementary file 1 [file DataSheet_1.docx]

Reduced serum calcium is associated with risk of retinopathy in non-diabetic individuals: the Chinese Multi-provincial Cohort Study

Jiangtao Li^1^, Dong Zhao^1^, Qiuju Deng^1^, Yongchen Hao^1^, Miao Wang^1^, Jiayi Sun^1^, Jun Liu^1^, Guandi Ren^2^, Huiqi Li^2^, Yue Qi^1*^, Jing Liu^1*^

^1^ Center for Clinical and Epidemiological Research, Beijing An Zhen Hospital, Capital Medical University, Beijing Institute of Heart, Lung and Blood Vessel Diseases, The Key Laboratory of Remodeling-Related Cardiovascular Diseases, Ministry of Education, Beijing Municipal Key Laboratory of Clinical Epidemiology, Beijing, China, Beijing, 100029, China

^2^ School of Information and Electronics, Beijing Institute of Technology, Beijing 100081, China

Additional files

[Table S1. Results of the normality test of continuous variables 2](#_Toc117172217)

[Table S2. Characteristics of the eligible participants for analyzing the association between serum calcium and retinopathy and those who were excluded 3](#_Toc117172218)

[Table S3. Characteristics of participants for analyzing the association between five-changes in serum calcium and retinopathy and those who were excluded due to lack of serum calcium in 2007 survey 4](#_Toc117172219)

[Table S4. Logistic regression analyses of the association between serum levels of calcium and retinopathy 5](#_Toc117172220)

[Table S5. Sensitive analyses on the association between serum levels of calcium and retinopathy 6](#_Toc117172221)

[Table S6. Logistic regression of the association between albumin-corrected calcium and retinopathy 7](#_Toc117172222)

[Figure S1. Flowchart of the study design and study population. 8](#_Toc117172223)

[Figure S2 The SMD of characteristics among IPTW unweighted and weighted samples. 9](#_Toc117172224)

[Figure S3. Partial correlations between serum calcium and other covariates controlling for age and sex. 10](#_Toc117172225)

[Figure S4. Restricted cubic spline plots for analyzing the risk of retinopathy based on calcium levels. 11](#_Toc117172226)

[Figure S5. The association between retinopathy prevalence and serum calcium stratified by clinical subgroups. 12](#_Toc117172227)

[Figure S6. Joint analysis for the association of calcium and metabolic risk factors with retinopathy. 13](#_Toc117172228)

**Table S1. Results of the normality test** **of continuous variables**

| **Characteristic** | ***P*** |
| --- | --- |
| Calcium, mmol/L | 0.208 |
| Albumin-corrected calcium, mmol/L | 0.327 |
| Age, years | <0.001 |
| Body mass index, kg/m^2^ | <0.001 |
| Systolic blood pressure, mmHg | 0.012 |
| Diastolic blood pressure, mmHg | 0.019 |
| Fasting blood glucose, mmol/L | <0.001 |
| Hemoglobin A1c, % | <0.001 |
| Total cholesterol, mmol/L | 0.200 |
| LDL-C, mmol/L | 0.104 |
| HDL-C, mmol/L | <0.001 |
| Triglyceride, mmol/L | <0.001 |
| Hs-CRP, mg/L | <0.001 |
| Albumin, g/L | 0.200 |
| eGFR, mL/min/1.73m^2^ | <0.001 |
| Magnesium, mmol/L | <0.001 |

eGFR, the estimated glomerular filtration rate; HDL-C, high-density lipoprotein cholesterol; hs-CRP, high-sensitivity C reactive protein; LDL-C, low-density lipoprotein cholesterol.

**Table S2. Characteristics of the eligible participants for analyzing the association between serum calcium and retinopathy and those who were excluded**

| **Characteristic ^a^** | **Included (n=1836)** | **Excluded (n=105)** | ***P*** |
| --- | --- | --- | --- |
| Age, years | 66 (59, 73) | 62 (59, 73) | 0.134 |
| Male, n (%) | 781 (42.5) | 40 (38.1) | 0.427 |
| Body mass index, kg/m^2^ | 24.2 (22.2, 26.4) | 24.7 (22.8, 26.2) | 0.293 |
| Smoking, n (%) | 213 (11.6) | 10 (13.7) | 0.718 |
| Systolic blood pressure, mmHg | 136.0 (125.7, 146.7) | 134.7 (122.5, 147.5) | 0.608 |
| Diastolic blood pressure, mmHg | 78.7 (72.7, 85.0) | 78.7 (73.3, 85.0) | 0.649 |
| Hypertension, n (%) | 1070 (58.3) | 60 (57.1) | 0.898 |
| Antihypertensive treatment, n (%) | 728 (39.7) | 38 (37.2) | 0.686 |
| Fasting blood glucose, mmol/L | 5.19 (4.89, 5.54) | 5.14 (4.87, 5.45) | 0.378 |
| Hemoglobin A1c, % | 5.6 (5.4, 5.9) | 5.6 (5.4, 5.9) | 0.329 |
| Pre-diabetes, n (%) | 130 (7.1) | 5 (5.4) | 0.693 |
| Total cholesterol, mmol/L | 5.28 ± 1.03 | 5.25 ± 0.89 | 0.724 |
| LDL-C, mmol/L | 3.09 ± 0.86 | 3.08 ± 0.78 | 0.917 |
| HDL-C, mmol/L | 1.31 (1.12, 1.53) | 1.36 (1.20, 1.58) | 0.109 |
| Triglyceride, mmol/L | 1.35 (0.95, 1.92) | 1.31 (0.92, 1.71) | 0.174 |
| Lipid-lowering treatment, n (%) | 400 (21.8) | 13 (13.0) | 0.054 |
| Hs-CRP, mg/L | 0.92 (0.47, 1.88) | 1.05 (0.46, 2.08) | 0.465 |
| Albumin, g/L | 45.31 ± 2.38 | 45.21 ± 2.59 | 0.711 |
| eGFR, mL/min/1.73m^2^ | 107.36 (94.98, 119.88) | 108.91 (97.29, 121.75) | 0.240 |
| Magnesium, mmol/L | 0.92 (0.88, 0.96) | 0.92 (0.88, 0.95) | 0.735 |

eGFR, the estimated glomerular filtration rate; HDL-C, high-density lipoprotein cholesterol; hs-CRP, high-sensitivity C reactive protein; LDL-C, low-density lipoprotein cholesterol. ^a^ Data are expressed as mean (standard deviation) for continuous variables in case of normal distributions and median (interquartile range) otherwise, and as number (percent) for categorical variables.

**Table S3. Characteristics of participants for analyzing the association between five-changes in serum calcium and retinopathy and those who were excluded due to lack of serum calcium in 2007 survey**

| **Characteristic ^a^** | **With calcium measurement in 2007 survey (n=1407)** | **Without calcium measurement in 2007 survey (n=429)** | ***P*** |
| --- | --- | --- | --- |
| Calcium, mmol/L | 2.44 ± 0.10 | 2.45 ± 0.09 | 0.077 |
| Albumin-corrected calcium, mmol/L | 2.33 ± 0.09 | 2.34 ± 0.09 | 0.059 |
| Age, years | 66 (59, 73) | 65 (59, 73) | 0.520 |
| Male, n (%) | 575 (40.9) | 206 (48.0) | 0.010 |
| Body mass index, kg/m^2^ | 24.2 (22.2, 26.4) | 24.2 (22.1, 26.7) | 0.889 |
| Smoking, n (%) | 148(10.5) | 65 (15.2) | 0.012 |
| Systolic blood pressure, mmHg | 135.7 (125.7, 146.0) | 136.7 (126.5, 148.5) | 0.106 |
| Diastolic blood pressure, mmHg | 78.3 (72.7, 84.7) | 79.7 (73.7, 86.0) | 0.028 |
| Hypertension, n (%) | 809 (57.5) | 261 (60.8) | 0.219 |
| Antihypertensive treatment, n (%) | 553 (39.5) | 175 (40.8) | 0.652 |
| Fasting blood glucose, mmol/L | 5.20 (4.89, 5.54) | 5.17 (4.90, 5.57) | 0.956 |
| Hemoglobin A1c, % | 5.6 (5.4, 5.9) | 5.6 (5.4, 5.8) | 0.234 |
| Pre-diabetes, n (%) | 92 (6.5) | 38 (8.9) | 0.107 |
| Total cholesterol, mmol/L | 5.28 ± 1.04 | 5.28 ± 0.98 | 0.957 |
| LDL-C, mmol/L | 3.09 ± 0.87 | 3.09 ± 0.84 | 0.932 |
| HDL-C, mmol/L | 1.31 (1.12, 1.53) | 1.31 (1.14, 1.53) | 0.823 |
| Triglyceride, mmol/L | 1.33 (0.93, 1.88) | 1.46 (0.98, 1.99) | 0.048 |
| Lipid-lowering treatment, n (%) | 311 (22.1) | 89 (20.7) | 0.593 |
| Hs-CRP, mg/L | 0.95 (0.48, 1.97) | 0.84 (0.43, 1.64) | 0.034 |
| Albumin, g/L | 45.31 ± 2.37 | 45.30 ± 2.43 | 0.926 |
| eGFR, mL/min/1.73m2 | 106.47 (94.51, 118.72) | 109.80 (96.26, 123.40) | 0.002 |
| Magnesium, mmol/L | 0.92 (0.88, 0.96) | 0.91 (0.87, 0.96) | 0.163 |
| History of cardiovascular disease, n (%) | 104 (7.4) | 27 (6.3) | 0.520 |

eGFR, the estimated glomerular filtration rate; HDL-C, high-density lipoprotein cholesterol; hs-CRP, high-sensitivity C reactive protein; LDL-C, low-density lipoprotein cholesterol. ^a^ Data are expressed as mean (standard deviation) for continuous variables in case of normal distributions and median (interquartile range) otherwise, and as number (percent) for categorical variables.

**Table S4. Logistic regression analyses of the association between serum levels of calcium and retinopathy**

| **Independent variables** | **Model 1** | | **Model 2** | |
| --- | --- | --- | --- | --- |
|  | **OR (95%CI)** | ***P*** | **OR (95%CI)** | ***P*** |
| Calcium quartiles | | | | |
| Quartile 4 (≥ 2.50 mmol/L) | Reference | | Reference | |
| Quartile 3 (2.44~2.50 mmol/L) | 1.05 (0.74, 1.50) | 0.785 | 1.05 (0.74, 1.49) | 0.808 |
| Quartile 2 (2.38~2.50 mmol/L) | 1.09 (0.76, 1.56) | 0.635 | 1.08 (0.75, 1.54) | 0.688 |
| Quartile 1 (< 2.38 mmol/L) | 1.58 (1.10, 2.27) | 0.015 | 1.55 (1.08, 2.23) | 0.018 |
| Age (years) | 1.05 (1.03, 1.07) | <0.001 | 1.05 (1.03, 1.07) | <0.001 |
| Female | 1.22 (0.92, 1.61) | 0.167 | 1.21 (0.92, 1.61) | 0.178 |
| Current smoking | 1.05 (0.69, 1.61) | 0.804 | 1.06 (0.69, 1.61) | 0.800 |
| Body mass index (kg/m^2^) | 1.01 (0.97, 1.05) | 0.560 | 1.01 (0.97, 1.06) | 0.512 |
| Systolic blood pressure strata | | | | |
| < 140 mmHg | Reference | | Reference | |
| 140-159 mmHg | 1.12 (0.85, 1.47) | 0.423 | 1.12 (0.85, 1.47) | 0.412 |
| ≥ 160 mmHg | 1.17 (0.74, 1.85) | 0.489 | 1.18 (0.75, 1.87) | 0.470 |
| Antihypertensive treatment | 0.97 (0.74, 1.28) | 0.829 | 0.97 (0.74, 1.28) | 0.847 |
| HDL-C, < 1.04/1.30 mmol/L | 1.02 (0.75, 1.38) | 0.918 | 1.02 (0.75, 1.39) | 0.899 |
| LDL-C, ≥ 3.40 mmol/L | 1.11 (0.85, 1.46) | 0.431 | 1.12 (0.85, 1.46) | 0.416 |
| Natural log-transformed triglyceride | 0.79 (0.60, 1.06) | 0.112 | 0.79 (0.59, 1.05) | 0.110 |
| Lipid-lowering treatment | 0.86 (0.62, 1.18) | 0.345 | 0.85 (0.62, 1.18) | 0.327 |
| Albumin (g/L) | 1.07 (1.01, 1.13) | 0.023 | 1.07 (1.01, 1.13) | 0.019 |
| eGFR, (mL/min/1.73m^2^) | 1.00 (0.99, 1.01) | 0.705 | 1 (1, 1.01) | 0.689 |
| Natural log-transformed hs-CRP | 1.03 (0.91, 1.18) | 0.611 | 1.03 (0.91, 1.18) | 0.606 |
| Magnesium, (0.10 mmol/L decrease) | 1.25 (1.03, 1.52) | 0.026 | 1.25 (1.03, 1.52) | 0.027 |
| Fasting blood glucose, ≥ 5.19 mmol/L | 1.13 (0.88, 1.45) | 0.330 | NA | NA |
| Hemoglobin A1c, ≥ 5.60 % | NA | NA | 1.05 (0.81, 1.36) | 0.710 |

CI, confidence interval; eGFR, the estimated glomerular filtration rate; HDL-C, high-density lipoprotein cholesterol; hs-CRP, high-sensitivity C reactive protein; LDL-C, low-density lipoprotein cholesterol; NA, not applicable; OR, odds ratio. Retinopathy was discerned by convolutional neural network.

**Table S5. Sensitive analyses on the association between serum levels of calcium and retinopathy**

| **Calcium (mmol/L)** | **Participants without pre-diabetes** | | **Participants without cardiovascular disease** | | **Participants with a normal range of serum calcium levels** | |
| --- | --- | --- | --- | --- | --- | --- |
|  | **OR (95%CI) ^a^** | ***P*** | **OR (95%CI) ^a^** | ***P*** | **OR (95%CI) ^a^** | ***P*** |
| Retinopathy discerned by CNN | | | | | | |
| ≥ 2.50 | Reference | | Reference | | Reference | |
| 2.44-2.50 | 0.95 (0.66, 1.37) | 0.770 | 1.03 (0.71, 1.49) | 0.887 | 1.07 (0.75, 1.53) | 0.694 |
| 2.38-2.44 | 1.01 (0.70, 1.46) | 0.956 | 1.02 (0.70, 1.48) | 0.930 | 1.12 (0.78, 1.60) | 0.552 |
| < 2.38 | 1.46 (1.00, 2.12) | 0.048 | 1.50 (1.02, 2.20) | 0.038 | 1.65 (1.15, 2.39) | 0.007 |
| Per SD decrease | 1.18 (1.01, 1.37) | 0.035 | 1.17 (1.01, 1.36) | 0.042 | 1.28 (1.10, 1.50) | 0.002 |
| Retinopathy discerned by the ophthalmologist | | | | | | |
| ≥ 2.50 | Reference | | Reference | | Reference | |
| 2.44-2.50 | 2.94 (1.30, 6.62) | 0.009 | 2.98 (1.31, 6.78) | 0.009 | 2.83 (1.29, 6.22) | 0.010 |
| 2.38-2.44 | 4.92 (2.25, 10.77) | <0.001 | 5.02 (2.29, 11.01) | <0.001 | 5.03 (2.38, 10.63) | <0.001 |
| < 2.38 | 7.59 (3.44, 16.74) | <0.001 | 8.44 (3.81, 18.72) | <0.001 | 8.23 (3.86, 17.53) | <0.001 |
| Per SD decrease | 2.09 (1.59, 2.73) | <0.001 | 2.29 (1.75, 3.01) | <0.001 | 2.41 (1.83, 3.19) | <0.001 |
| Retinopathy discerned by either CNN or the ophthalmologist | | | | | | |
| ≥ 2.50 | Reference | | Reference | | Reference | |
| 2.44-2.50 | 1.18 (0.83, 1.67) | 0.367 | 1.27 (0.89, 1.81) | 0.193 | 1.30 (0.93, 1.84) | 0.128 |
| 2.38-2.44 | 1.30 (0.91, 1.86) | 0.144 | 1.33 (0.93, 1.91) | 0.119 | 1.42 (1.01, 2.01) | 0.045 |
| < 2.38 | 2.03 (1.41, 2.91) | <0.001 | 2.16 (1.50, 3.12) | <0.001 | 2.29 (1.61, 3.27) | <0.001 |
| Per SD decrease | 1.35 (1.17, 1.56) | <0.001 | 1.37 (1.18, 1.58) | <0.001 | 1.48 (1.27, 1.74) | <0.001 |

CI, confidence interval; CNN, convolutional neural network; SD, standard deviation; OR, odds ratio. ^a^ ORs were calculated by logistic regressions after adjusting for age, sex, body mass index, smoking, systolic blood pressure, antihypertensive treatment, high-density lipoprotein cholesterol, low-density lipoprotein cholesterol, natural log-transformed triglyceride, lipid-lowering treatment, fasting blood glucose, natural log-transformed high-sensitivity C-reactive protein, albumin, the estimated glomerular filtration rate, and serum magnesium.

**Table S6. Logistic regression of the association between albumin-corrected calcium and retinopathy**

| **Albumin-corrected calcium (mmol/L)** | **Unadjusted** | | **Model 1 ^a^** | | **Model 2 ^b^** | |
| --- | --- | --- | --- | --- | --- | --- |
|  | **OR (95%CI)** | ***P*** | **OR (95%CI)** | ***P*** | **OR (95%CI)** | ***P*** |
| Retinopathy discerned by CNN | | | | | | |
| ≥ 2.40 | Reference | | Reference | | Reference | |
| 2.34-2.40 | 0.86 (0.60, 1.23) | 0.411 | 0.93 (0.64, 1.34) | 0.686 | 0.93 (0.64, 1.33) | 0.674 |
| 2.27-2.34 | 1.03 (0.73, 1.45) | 0.852 | 1.25 (0.87, 1.79) | 0.227 | 1.22 (0.86, 1.75) | 0.269 |
| < 2.27 | 1.38 (0.99, 1.91) | 0.057 | 1.83 (1.28, 2.61) | 0.001 | 1.79 (1.25, 2.55) | 0.001 |
| Per SD decrease | 1.13 (0.99, 1.28) | 0.075 | 1.31 (1.13, 1.52) | <0.001 | 1.30 (1.12, 1.50) | 0.001 |
| Retinopathy discerned by the ophthalmologist | | | | | | |
| ≥ 2.40 | Reference | | Reference | | Reference | |
| 2.34-2.40 | 2.78 (1.22, 6.34) | 0.015 | 2.95 (1.28, 6.76) | 0.011 | 2.95 (1.27, 6.71) | 0.011 |
| 2.27-2.34 | 3.08 (1.37, 6.92) | 0.007 | 3.56 (1.56, 8.13) | 0.003 | 3.45 (1.51, 7.86) | 0.003 |
| < 2.27 | 7.49 (3.53, 15.92) | <0.001 | 9.40 (4.29, 20.59) | <0.001 | 9.05 (4.14, 19.80) | <0.001 |
| Per SD decrease | 2.14 (1.69, 2.71) | <0.001 | 2.45 (1.87, 3.21) | <0.001 | 2.42 (1.85, 3.17) | <0.001 |
| Retinopathy discerned by either CNN or the ophthalmologist | | | | | | |
| ≥ 2.40 | Reference | | Reference | | Reference | |
| 2.34-2.40 | 1.03 (0.73, 1.45) | 0.853 | 1.13 (0.79, 1.60) | 0.504 | 1.12 (0.79, 1.59) | 0.520 |
| 2.27-2.34 | 1.19 (0.85, 1.66) | 0.309 | 1.47 (1.04, 2.09) | 0.030 | 1.44 (1.02, 2.03) | 0.041 |
| < 2.27 | 1.84 (1.34, 2.52) | <0.001 | 2.52 (1.78, 3.55) | <0.001 | 2.45 (1.74, 3.46) | <0.001 |
| Per SD decrease | 1.28 (1.13, 1.45) | <0.001 | 1.51 (1.31, 1.75) | <0.001 | 1.49 (1.29, 1.72) | <0.001 |

CI, confidence interval; CNN, convolutional neural network; SD, standard deviation; OR, odds ratio. ^a^ Adjusted for age, sex, body mass index, smoking, systolic blood pressure, antihypertensive treatment, high-density lipoprotein cholesterol, low-density lipoprotein cholesterol, natural log-transformed triglyceride, lipid-lowering treatment, fasting blood glucose, natural log-transformed high-sensitivity C-reactive protein, albumin, the estimated glomerular filtration rate, and serum magnesium. ^b^ Model 1 + hemoglobin A1c substituting for fasting blood glucose.


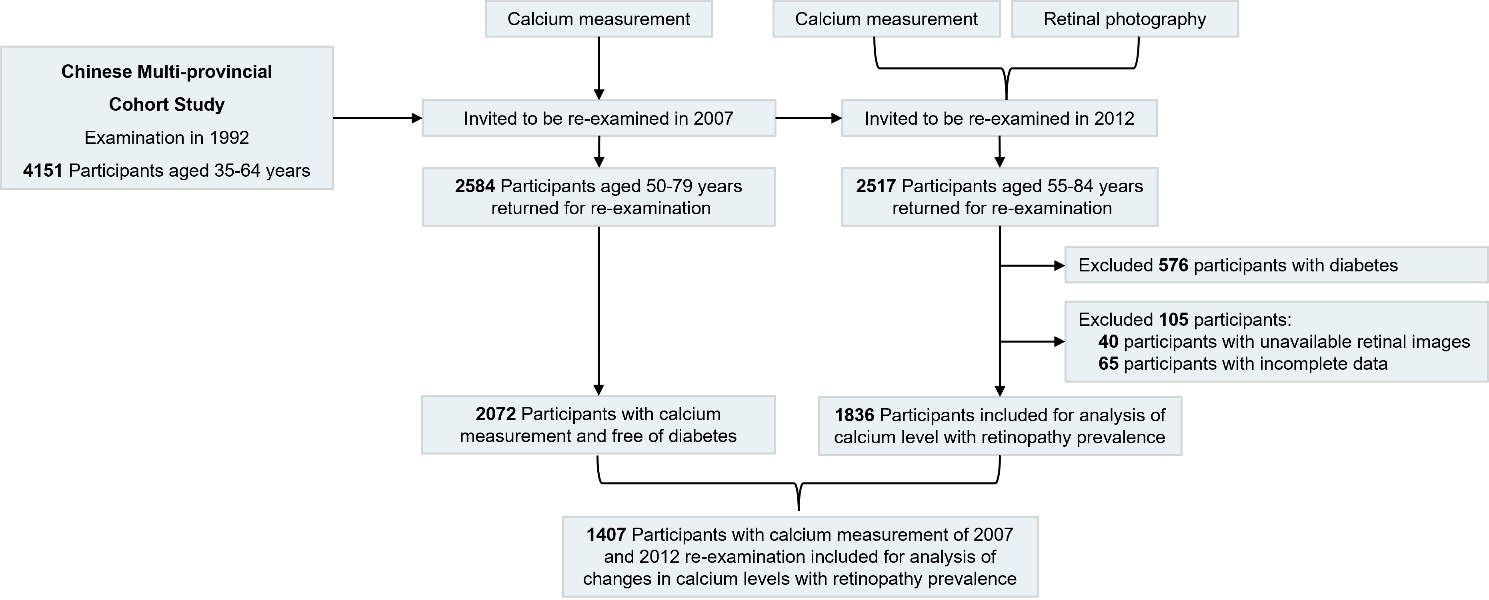


**Figure S1. Flowchart of the study design and study population.**


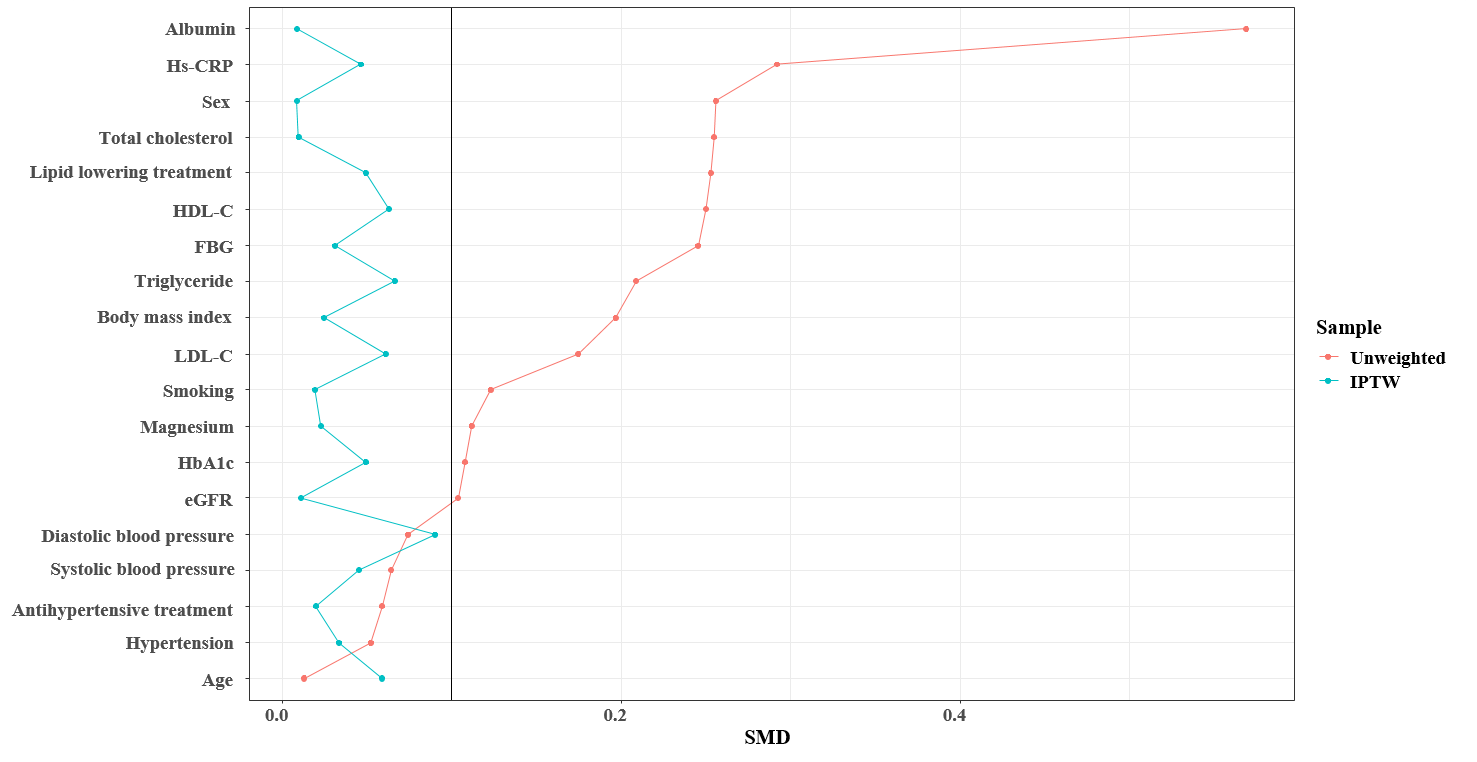


**Figure S2 The SMD of characteristics among IPTW unweighted and weighted samples.**

eGFR, the estimated glomerular filtration rate; FBG, fasting blood glucose; HbA1c, hemoglobin A1c; HDL-C, high-density lipoprotein cholesterol; hs-CRP, high-sensitivity C reactive protein; IPTW, inverse probability of treatment weighting; LDL-C, low-density lipoprotein cholesterol; SMD, standardized mean differences.

**Figure S3.** **Partial correlations between serum calcium and other covariates** **controlling for age and sex**.

BMI, body mass index; DBP, diastolic blood pressure; eGFR, the estimated glomerular filtration rate; FBG, fasting blood glucose; HbA1c, hemoglobin A1c; HDL-C, high-density lipoprotein cholesterol; hs-CRP, high-sensitivity C reactive protein; LDL-C, low-density lipoprotein cholesterol; SBP, systolic blood pressure; TC, total cholesterol; TG, triglyceride.


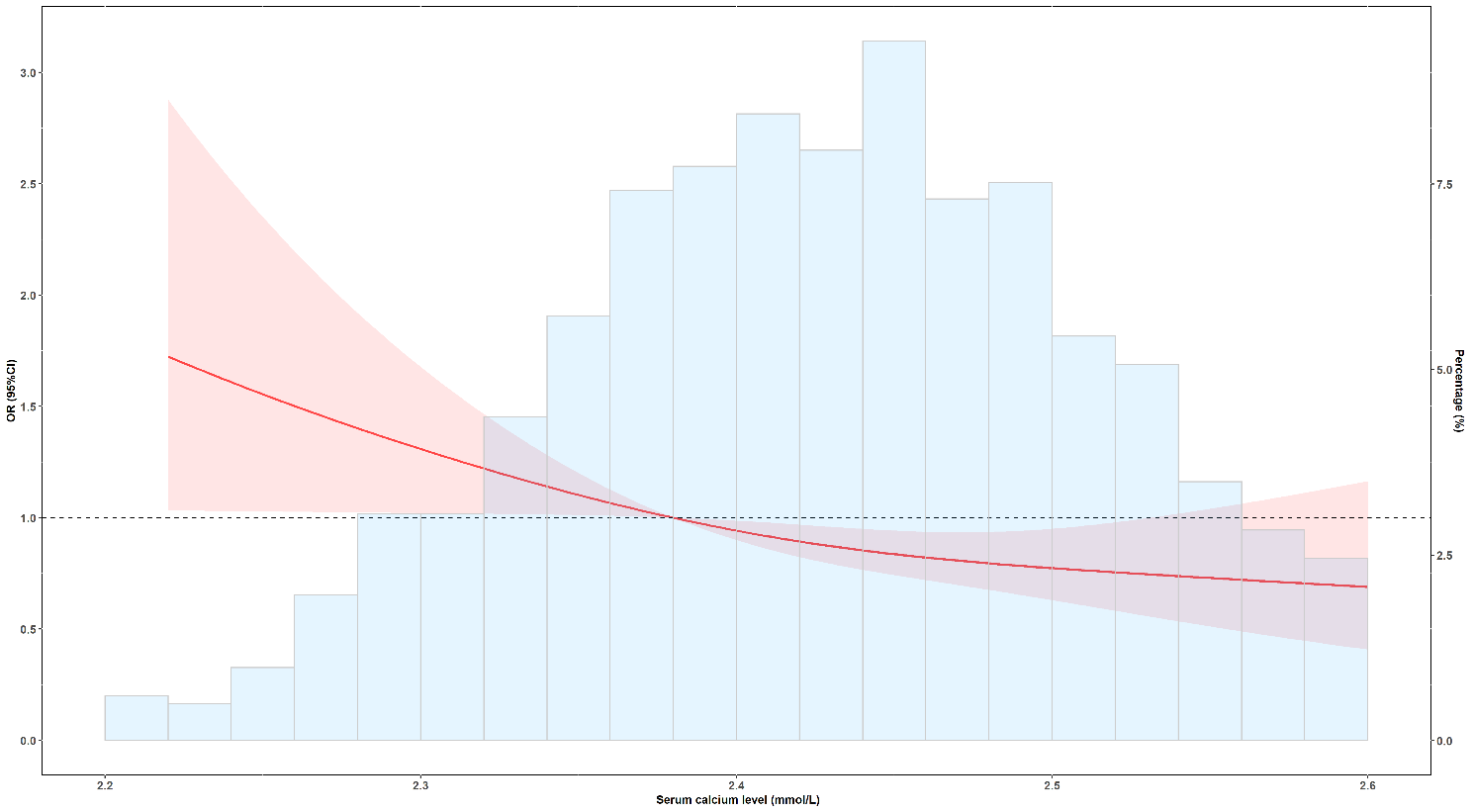


**Figure S4. Restricted cubic spline plots for analyzing the risk of retinopathy based on calcium levels.**

CI, confidence interval; OR, odds ratio. The background histograms (light blue color) represent the percent density distribution of serum calcium in the study population (right Y-axis). The heavy central red line represents the estimated adjusted odds ratios, with shaded ribbons denoting 95% confidence intervals. The horizontal dotted lines represent the odds ratio of 1.0. The reference point was set at a serum calcium level of 2.38 mmol/L (the first knots, 25th percentile of serum calcium). The P-value for nonlinear was 0.47. Retinopathy was discerned by convolutional neural network.


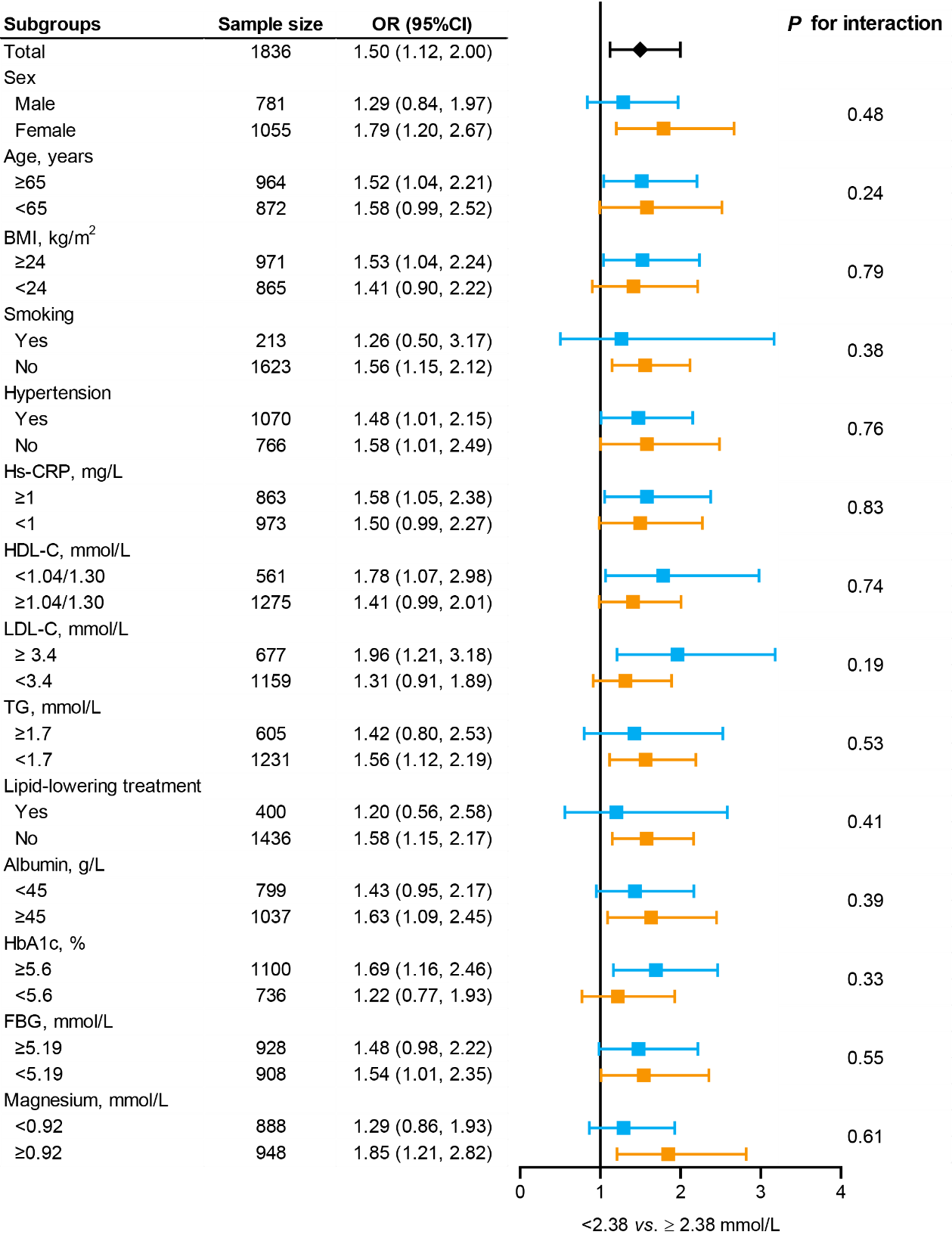


**Figure S5. The association between retinopathy prevalence and serum calcium stratified by clinical subgroups.**

BMI, body mass index; CI, confidence interval; eGFR, the estimated glomerular filtration rate; FBG, fasting blood glucose; HDL-C, high-density lipoprotein cholesterol; hs-CRP, high-sensitivity C reactive protein; LDL-C, low-density lipoprotein cholesterol; OR, odds ratio; TG, triglyceride. ORs were calculated by logistic regressions after adjusting for age, sex, BMI, smoking, systolic blood pressure, antihypertensive treatment, HDL-C, LDL-C, natural log-transformed TG, lipid-lowering treatment, FBG, log-transformed hs-CRP, albumin, eGFR, and serum magnesium, except where an adjusting variable was itself being tested. *P* for interaction was assessed by including a multiplicative interaction term in the logistic regression models. Retinopathy was discerned by convolutional neural network.


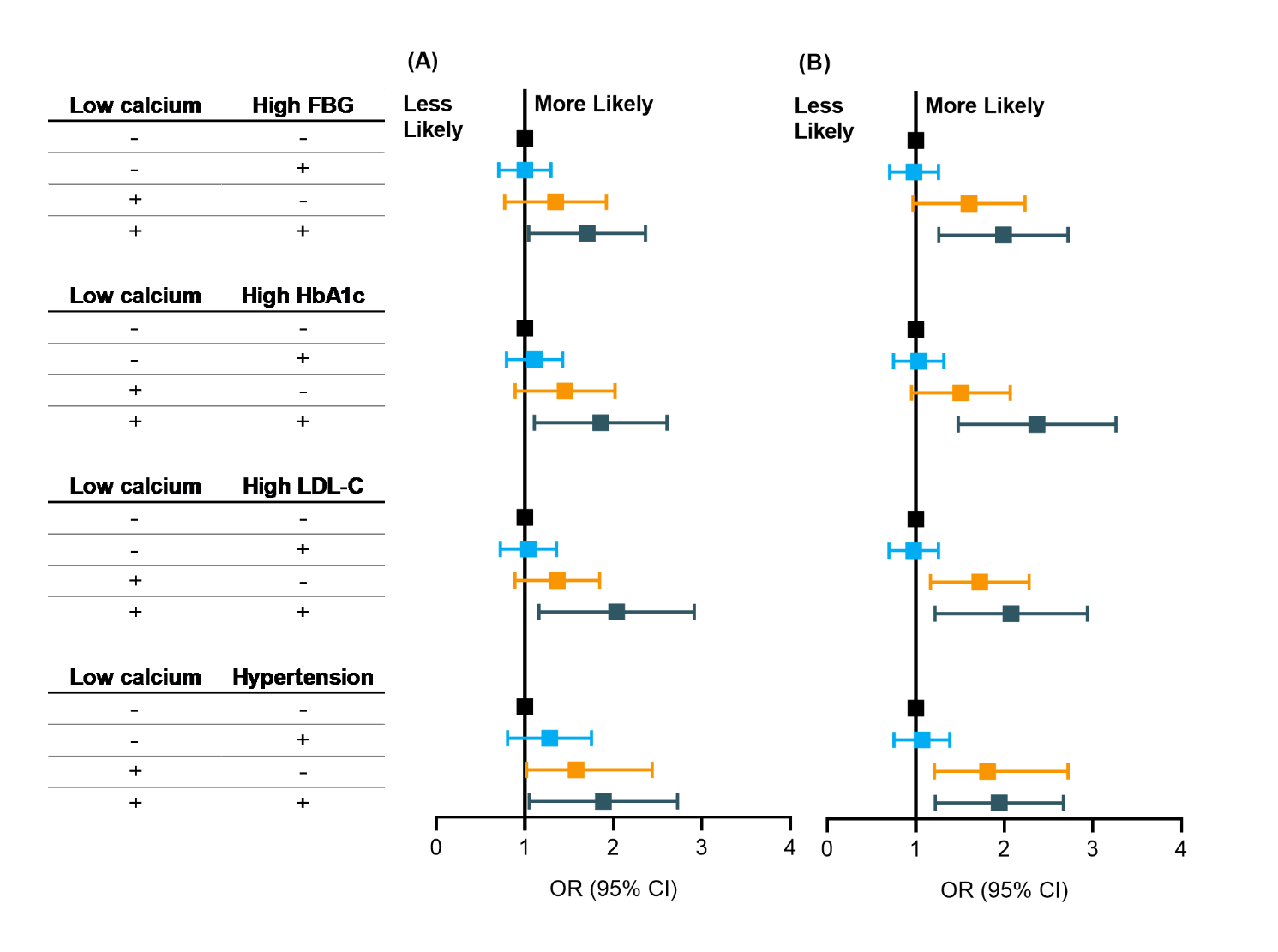


**Figure S6. Joint analysis for the association of calcium and metabolic risk factors with retinopathy.**

(A), retinopathy discerned by CNN. (B), retinopathy discerned by CNN or ophthalmologist. Low calcium: calcium < 2.38 mmol/L. High FBG: FBG ≥ 5.19 mmol/L. High HbA1c: HbA1c ≥ 5.6%. High LDL-C: LDL-C ≥ 3.4 mmol/L. Hypertension: systolic blood pressure ≥ 140 mmHg, diastolic blood pressure ≥ 90 mmHg, and/or antihypertensive treatment in the last two weeks. CI, confidence interval; CNN, convolutional neural network; FBG, fasting blood glucose; HbA1c, hemoglobin A1c; HDL-C, high-density lipoprotein cholesterol; LDL-C, low-density lipoprotein cholesterol; OR, odds ratio.
